# The Genetic Profile of Large B-Cell Lymphomas Presenting in the Ocular Adnexa

**DOI:** 10.3390/ijms25063094

**Published:** 2024-03-07

**Authors:** Stine Dahl Vest, Patrick Rene Gerhard Eriksen, Fleur A. de Groot, Ruben A. L. de Groen, Anne H. R. Kleij, Marina Knudsen Kirkegaard, Peter Kamper, Peter Kristian Rasmussen, Christian von Buchwald, Peter de Nully Brown, Jens Folke Kiilgaard, Joost S. P. Vermaat, Steffen Heegaard

**Affiliations:** 1 Department of Pathology, Copenhagen University Hospital—Rigshospitalet, 2100 Copenhagen, Denmark; 2 Department of Ophthalmology, Copenhagen University Hospital—Rigshospitalet, 2100 Copenhagen, Denmark; 3 Department of Otorhinolaryngology, Head and Neck Surgery and Audiology, Copenhagen University Hospital—Rigshospitalet, 2100 Copenhagen, Denmark; 4 Department of Hematology, Leiden University Medical Center, 2333 ZA Leiden, The Netherlands; 5 Department of Hematology, Aarhus University Hospital, 8200 Aarhus, Denmark; 6 Department of Hematology, Copenhagen University Hospital—Rigshospitalet, 2100 Copenhagen, Denmark

**Keywords:** ocular adnexal lymphoma, diffuse large B-cell lymphoma, high-grade B-cell lymphoma, genetics, mutation, next-generation sequencing, copy number variation

## Abstract

To provide insights into targetable oncogenic pathways, this retrospective cohort study investigated the genetic profile of 26 patients with diffuse large B-cell lymphoma, not otherwise specified (DLBCL-NOS), and two patients with high-grade B-cell lymphoma with *MYC* and *BCL2* rearrangements (HGBCL) presenting in the ocular adnexa. Pathogenic variants and copy number variations in 128 B-cell lymphoma-relevant genes were analyzed by targeted next-generation sequencing. Genetic subtypes were determined with the LymphGen algorithm. Primary ocular adnexal DLBCL-NOS constituted 50% (n = 14) and was generally characterized by non-germinal center B-cell origin (non-GCB) (n = 8, 57%), and LymphGen MCD subtype (n = 5, 36%). Primary ocular adnexal DLBCL-NOS presented pathogenic variants in genes involved in NF-κB activation and genes which are recurrently mutated in other extranodal lymphomas of non-GCB origin, including *MYD88* (n = 4, 29%), *CD79B* (n = 3, 21%), *PIM1* (n = 3, 21%), and *TBL1XR1* (n = 3, 21%). Relapsed DLBCL-NOS presenting in the ocular adnexa (n = 6) were all of non-GCB origin and frequently of MCD subtype (n = 3, 50%), presenting with a similar genetic profile as primary ocular adnexal DLBCL-NOS. These results provide valuable insights into genetic drivers in ocular adnexal DLBCL-NOS, offering potential applications in future precision medicine.

## 1. Introduction

Diffuse large B-cell lymphoma, not otherwise specified (DLBCL-NOS) represents the most common lymphoma subtype, constituting approximately 25–35% of all non-Hodgkin lymphomas [1]. Patient survival has improved significantly after the addition of anti-CD20 immunotherapy, namely rituximab, to the standard chemotherapy regimen of cyclophosphamide, doxorubicin, vincristine, and prednisone (R-CHOP), achieving long-lasting remissions in approximately 60% of patients [2]. Patient prognosis is associated with both clinical features, as defined by the International Prognostic Index (IPI) [3,4], and molecular profiles, including cell-of-origin (COO) [1]. Recently, the WHO lymphoma classification was updated, and high-grade B-cell lymphoma with *MYC* and *BCL2* gene rearrangements (HGBCL) was designated as its own subtype, with an inferior prognosis compared to DLBCL-NOS [5]. However, DLBCL-NOS is still a very heterogeneous disease with variable prognosis, and currently, the genetic landscape is being explored to find targetable oncogenic pathways for the development of new precision medicine for patients that do not achieve durable remission on R-CHOP [6,7,8,9,10]. This has led to the discovery of approximately 150 putative driver genes that are recurrently mutated or targets of copy number variations (CNV) [6,7,8,9,10]. Furthermore, recent studies have defined several genetic subgroups in DLBCL-NOS based on genetic mutational clusters with the involvement of subtype-specific biological pathways and a difference in patient outcome [7,8,9,10]. Of these, the LymphGen study has made its algorithm publicly available, enabling genetic subtyping in future genetic DLBCL-NOS studies and clinical trials for precision medicine [7]. Treatment allocation based on the molecular and genetic profile is thus now being incorporated into the first clinical DLBCL-NOS trials [11,12,13].

In up to 40% of cases, DLBCL-NOS manifests solely as an extranodal disease [1], and the genetic landscape differs between the primary extranodal locations and compared to nodal DLBCL-NOS [14,15]. Although rare, DLBCL-NOS and other large B-cell lymphomas (LBCL) can present as extranodal disease in the ocular adnexa, i.e., the orbit, conjunctiva, eyelid, lacrimal gland, and lacrimal draining system. Approximately 10% of lymphomas in the ocular adnexa are LBCL (OA-LBCL), with the majority being DLBCL-NOS [16,17,18,19], although HGBCL also accounts for a minor proportion of cases [20]. Recently, our research group investigated *MYC*, *BCL2*, and *BCL6* rearrangements as well as pathogenic variants of *MYD88* and *CD79B* in our Danish cohort of patients with DLBCL-NOS and HGBCL involving the ocular adnexa (n = 34), and showed that *MYD88* pathogenic variants were present in 29% of the patients [20]. However, the remaining genetic landscape has not been fully elucidated, as previous targeted next-generation sequencing (NGS) studies on DLBCL-NOS involving the ocular adnexa have only included seven and three patients, respectively [21,22]. This lack of knowledge may hinder the translation of new targeted therapies from clinical trials designed for DLBCL-NOS to their potential application in OA-LBCL patients. Therefore, the present study aimed at a comprehensive characterization of the genetic profile of DLBCL-NOS and HGBCL presenting in the ocular adnexa through targeted NGS analysis of pathogenic variants and CNVs in 128 highly lymphoma relevant genes, and classification of patients into clinically relevant genetic subtypes through application of the LymphGen algorithm.

## 2. Results

**Figure 1:**
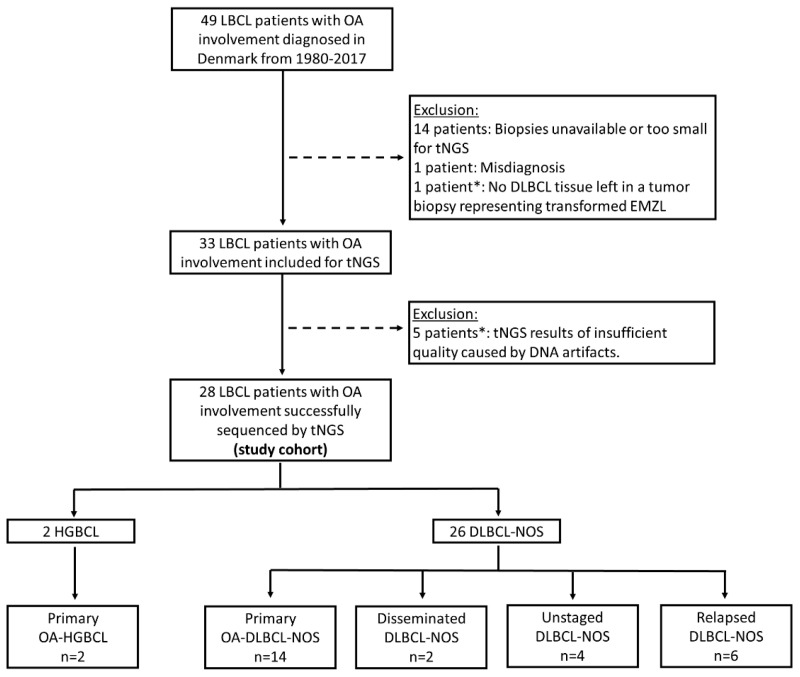
Flowchart illustrating the inclusion of patients in the study and patient subgroups by disease category. * Marks patients excluded in the present study in comparison to our previously published OA-LBCL cohort (n = 34) [20]. Abbreviations: DLBCL-NOS: diffuse large B-cell lymphoma, not otherwise specified; EMZL: extranodal marginal zone lymphoma; HGBCL: high-grade B-cell lymphoma with *MYC* and *BCL2* rearrangements; LBCL: large B-cell lymphoma; OA: ocular adnexal; tNGS: targeted next-generation sequencing.

A total of 49 LBCL patients with ocular adnexal involvement diagnosed in Denmark between 1980 and 2017 were identified as part of a previous nationwide study [18]. Of these, we excluded 14 patients due to unavailable or small biopsies, 1 patient due to misdiagnosis, and 1 patient due to the lack of DLBCL tumor tissue in a transformed extranodal marginal zone lymphoma biopsy. This resulted in 33 patients eligible for targeted NGS, of whom the results of 5 patients were excluded due to insufficient quality, presumably caused by formalin-fixation-induced DNA artifacts. This left a final study population of 28 patients with LBCL involving the ocular adnexa (Figure 1). These patients have previously been published as part of a prior study (n = 34) investigating *MYC*, *BCL2*, and *BCL6* rearrangements, pathogenic variants of *MYD88* and *CD79B,* and double-expressor lymphoma [20].

### 2.1. Pathological Features

**Figure 2:**
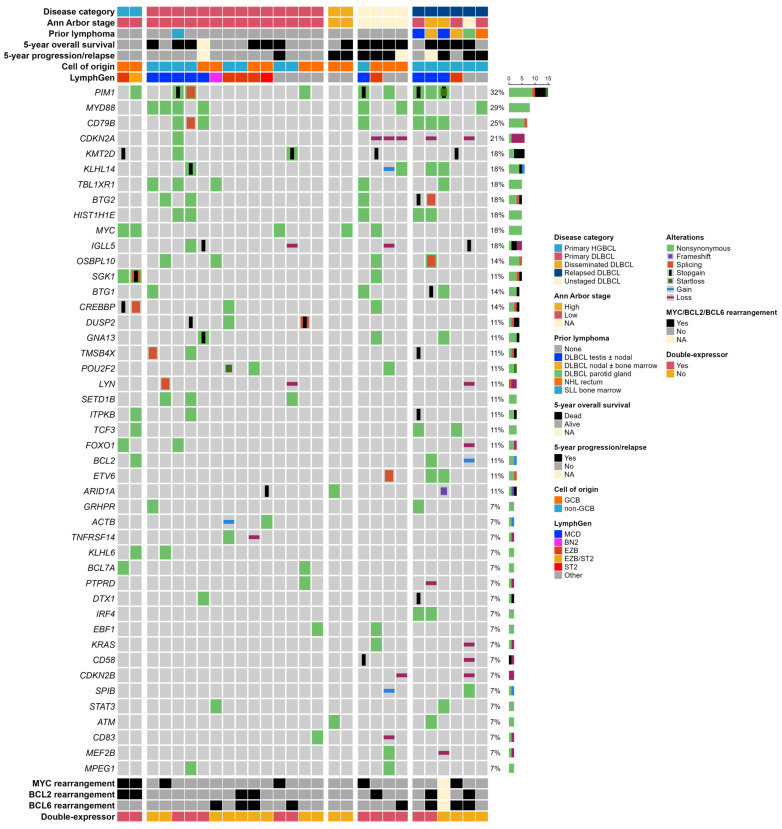
Oncoprint displaying recurrently altered genes (gene alteration frequency > 5%) in 26 patients with DLBCL-NOS involving the ocular adnexa and 2 patients with ocular adnexal HGBCL as well as clinical and molecular characteristics. Every row represents a specific gene or clinical characteristic, and every column represents a patient. The percentage of patients with alterations in the denoted gene is shown on the right side. Patients are divided according to disease category. Clinical patient characteristics are shown on the top, including disease category, Ann Arbor stage, prior lymphoma diagnosis, prognosis by all-cause mortality and progression/relapse of disease within 5 years from ocular adnexal LBCL diagnosis, as well as molecular characteristics such as cell-of-origin by Hans’ classification and LymphGen genetic subgrouping. Rearrangements of *MYC*, *BCL2*, and *BCL6* as well as double-expressor lymphoma with overexpression of both MYC and BCL2 are shown at the bottom. Color codes are depicted on the right side. Abbreviations: DLBCL: diffuse large B-cell lymphoma; GCB: germinal center B-cell; HGBCL: high-grade B-cell lymphomas with *MYC* and *BCL2* rearrangements; NA: not available; NHL: non-Hodgkin lymphoma, not otherwise specified; SLL: small lymphocytic lymphoma.

According to the fifth edition of the WHO Classification of Tumours of Haematopoietic and Lymphoid Tissues [5], 26 patients (93%) were classified as DLBCL-NOS, and 2 patients (7%) were classified as HGBCL with *MYC* and *BCL2* rearrangements. Based on expert hematopathological review, none of the tumor samples contained an extranodal marginal zone lymphoma component. Rearrangements of *MYC*, *BCL2*, and *BCL6*, and double-expressor lymphoma with immunohistochemical overexpression of both MYC and BCL2 were evaluated as part of a previously published study [20]; the results are displayed in Figure 2.

### 2.2. Patient Clinical Characteristics

The two patients with HGBCL were categorized as primary ocular adnexal HGBCL (primary OA-HGBCL), as defined by localized ocular adnexal disease (Ann Arbor stage IE-IIE) and no prior history of lymphoma (Figure 1, Table 1). Of the 26 patients with DLBCL-NOS, 14 patients (54%) presented with primary localized DLBCL-NOS of the ocular adnexa (Ann Arbor stage IE-IIE) (primary OA-DLBCL-NOS), 2 patients (8%) had disseminated DLBCL-NOS with ocular adnexal involvement at the time of diagnosis (Ann Arbor stage IV) (disseminated DLBCL-NOS), and 4 patients (15%) with ocular adnexal DLBCL-NOS were not staged due to frailty/comorbidities, but did not have any prior history of lymphoma (unstaged DLBCL-NOS) (Figure 1, Table 1). Two of the primary OA-DLBCL-NOS patients had discordant bone marrow involvement with small lymphocytic lymphoma (SLL) infiltration, but were still considered primary OA-DLBCL-NOS with low-stage disease (Ann Arbor stage IE). In one of these two patients, SLL bone marrow involvement was diagnosed before ocular adnexal DLBCL-NOS, but with no transformation in the bone marrow biopsy at the time of ocular adnexal DLBCL-NOS diagnosis. The remaining six DLBCL-NOS patients (6 of 26, 23%) had a prior lymphoma diagnosis, and ocular adnexal DLBCL-NOS involvement was part of their relapsed disease (relapsed DLBCL-NOS) (Figure 1, Table 1). Relapsed DLBCL-NOS with secondary ocular adnexal involvement thus included two patients with previous testicular DLBCL with/without nodal involvement, two patients with previous nodal DLBCL-NOS with/without bone marrow involvement, one patient with previous DLBCL-NOS of the parotid gland, and one patient with previous non-Hodgkin lymphoma, not otherwise specified, of the rectum. Patient categories are displayed in Figure 1, whereas patient characteristics, treatment, and follow-up information are listed in Table 1.

**Table 1:** Clinical characteristics of 28 LBCL patients with ocular adnexal involvement.

	All n = 28	HGBCL n = 2	DLBCL-NOS n = 20	Relapsed DLBCL-NOS ^8,9^ n = 6
**Age ^1^ (years)**	74 (69–83)	71 (69–74)	75 (71–83)	71 (66–84)
**Sex**				
Male	13 (46)	0 (0)	10 (50)	3 (50)
Female	15 (54)	2 (100)	10 (50)	3 (50)
**OA-LBCL location**				
Orbit ± other OA location ^2^	25 (89)	2 (100)	18 (90)	5 (83)
Lacrimal sac	2 (7)	0 (0)	2 (10)	0 (0)
Eyelid	1 (4)	0 (0)	0 (0)	1 (17)
**Cell-of-origin**				
GCB	13 (46)	2 (100)	11 (55)	0 (0)
Non-GCB	15 (54)	0 (0)	9 (45)	6 (100)
**Ann Arbor stage at OA-LBCL diagnosis**				
IE-IIE	19 (68)	2 (100)	14 (70) ^7^	3 (50) ^10^
IV	4 (14)	0 (0)	2 (10)	2 (33)
Unstaged	5 (18)	0 (0)	4 (20)	1 (17)
**Initial treatment of OA-LBCL**				
Rituximab + chemotherapy ± RT ^3^	13 (46)	1 (50)	10 (50)	2 (33)
RT ± prednisolone ^4^	9 (32)	0 (0)	7 (35)	2 (33)
Chemotherapy ± RT ^5^	3 (11)	1 (50)	0 (0)	2 (33)
No treatment ^6^	3 (11)	0 (0)	3 (15)	0 (0)
**Recurrence or progression within 5 years of OA-LBCL diagnosis**				
Yes	9 (32)	0 (0)	6 (30)	3 (50)
No	16 (57)	2 (100)	12 (60)	2 (33)
Unknown	3 (11)	0 (0)	2 (10)	1 (17)
**Disease status at 5-year follow-up from OA-LBCL diagnosis**				
Alive with complete remission	12 (43)	2 (100)	8 (40)	2 (33)
Dead from lymphoma	9 (32)	0 (0)	6 (30)	3 (50)
Dead from other cause	4 (14)	0 (0)	3 (15)	1 (17)
Dead from unknown cause	2 (7)	0 (0)	2 (10)	0 (0)
Unknown vital status	1 (4)	0(0)	1 (5)	0 (0)
**Time to last follow-up from OA-LBCL diagnosis, months ^1^**	30 (8–107)	193 (170–216)	25 (3–90)	21 (8–83)

^1^ Displayed as median and interquartile range. ^2^ Orbital lymphoma ± involvement of other ocular adnexal structures such as the conjunctiva, eyelid, or lacrimal gland. ^3^ Eleven patients received R-CHOP of 3–6 doses. Two patients received only 1 dose of R-CHOP due to comorbidities or death precluding further chemotherapy. Six patients further received high-dose MTX or intrathecal MTX in addition to R-CHOP. Ten patients received RT in addition to chemotherapy. ^4^ One patient received palliative intended RT (relapsed DLBCL-NOS), three patients received RT ± prednisolone due to comorbidities/frailty precluding chemotherapy, and five patients received RT ± prednisolone, as this treatment regimen has been used historically for low-stage DLBCL-NOS. ^5^ One patient received CHOP and high-dose MTX, one patient received CVP and RT (relapsed DLBCL-NOS), and one patient received high-dose MTX, high-dose cytarabine, prednisolone, and RT (relapsed DLBCL-NOS). ^6^ Two patients died before the initiation of treatment, and one patient was not treated due to age and frailty. ^7^ Two patients had discordant bone marrow involvement with SLL infiltration but were still considered primary OA-DLBCL-NOS and Ann Arbor stage IE. ^8^ Relapsed DLBCL-NOS includes patients with prior lymphoma with secondary relapse to the ocular adnexa. Primary lymphoma diagnoses prior to OA-LBCL relapse included testicular DLBCL ± nodal involvement (n = 2), DLBCL-NOS in the parotid gland (n = 1), nodal DLBCL-NOS ± bone marrow involvement (n = 2), and NHL in the rectum (n = 1). The median time from primary lymphoma diagnosis to OA-LBCL diagnosis was 43 months (IQR 12–101 months). ^9^ Primary treatment of relapsed patients prior to OA-LBCL relapse included: two patients with prior nodal DLBCL-NOS treated with R-CEOP or CHOP + CEOP, two patients with prior testicular DLBCL treated with CHOP or R-CHOP in combination with RT and CNS prophylaxis (high-dose methotrexate or intrathecal cytarabine), one patient with NHL rectum treated with surgical resection, and one patient with parotid gland DLBCL-NOS treated with RT. ^10^ One patient had discordant indolent lymphoma involvement of the bone marrow. Abbreviations: CEOP: cyclophosphamide, etoposide, vincristine, prednisolone; CHOP: cyclophosphamide, doxorubicin, vincristine, prednisolone; CVP: cyclophosphamide, vincristine, prednisolone; DLBCL-NOS: diffuse large B-cell lymphoma, not otherwise specified; GCB: germinal center B-cell; HGBCL: high-grade B-cell lymphomas with *MYC* and *BCL2* rearrangements; LBCL: large B-cell lymphoma; MTX: methotrexate; NHL: non-Hodgkin lymphoma; OA: ocular adnexal; OA-LBCL: Ocular adnexal large B-cell lymphoma; R: rituximab; RT: radiotherapy.

### 2.3. Cell-of-Origin

Cell-of-origin by Hans’ algorithm [23] revealed 11 of 26 (42%) DLBCL-NOS tumors to be of germinal center B-cell (GCB) origin and 15 of 26 (58%) of non-GCB origin. Patients with primary OA-DLBCL-NOS also had a slightly higher proportion of non-GCB cases (8 of 14, 57%) than GCB cases (6 of 14, 43%) (Figure 2). All cases of relapsed DLBCL-NOS with secondary ocular adnexal involvement were of non-GCB origin (n = 6, 100%) (Figure 2, Table 1). Both primary OA-HGBCL were of GCB origin (n = 2, 100%) (Figure 2, Table 1).

### 2.4. Targeted Next-Generation Sequencing

In total, 28 OA-LBCL samples successfully underwent targeted NGS of 128 genes. Of these, 22 samples (79%) were sequenced successfully in the first run, whereas 6 samples (21%) were re-sequenced with a DNA repair kit due to deamination artefacts caused by formalin fixation.

Pathogenic variants were identified in 71 genes, and CNVs were identified in 25 genes. Genes displaying pathogenic variants or CNVs in a minimum of two patients are presented in Figure 2, and all sequenced genes and alterations are displayed in Supplementary Figure S1. The average read count of pathogenic variants was 1122 (range 106–7985). The patients harbored a median of five genes with pathogenic variants per individual (interquartile range (IQR): 2–8.25), and a median of six genes (IQR: 3–10.25) with either pathogenic variants or CNVs per individual. One patient tumor sample did not present any pathogenic variants or CNVs, which was validated by a second targeted NGS analysis of this sample.

Primary OA-DLBCL-NOS frequently exhibited pathogenic variants in *MYD88* (4 of 14, 29%), *CD79B* (3 of 14, 21%), *PIM1* (3 of 14, 21%), *TBL1XR1* (3 of 14, 21%), *SETD1B* (3 of 14, 21%), and *DUSP2* (3 of 14, 21%) (Figure 2). Primary OA-DLBCL-NOS harbored a median of 4.5 genes (IQR: 3–6) with pathogenic variants or CNVs.

Relapsed DLBCL-NOS involving the ocular adnexa frequently presented pathogenic variants in *PIM1* (3 of 6, 50%), *CD79B* (3 of 6, 50%), *MYD88* (2 of 6, 33%), *KLHL14* (2 of 6, 33%), *BTG1* (2 of 6, 33%), *BTG2* (2 of 6, 33%), *HIST1H1E* (2 of 6, 33%), *TCF3* (2 of 6, 33%), *ETV6* (2 of 6, 33%), and *IRF4* (2 of 6, 33%). A copy number loss of *CDKN2A* was found in two of six relapsed DLBCL-NOS patients (33%) (Figure 2). Relapsed DLBCL-NOS with ocular adnexal involvement harbored a median of 11 genes (IQR: 6.5–12.5) with pathogenic variants or CNVs.

Both primary OA-HGBCL presented pathogenic variants in *MYC* (2 of 2, 100%), *SGK1* (2 of 2, 100%), and *CREBBP* (2 of 2, 100%) (Figure 2). No pathogenic variants of *TP53* or *CARD11* were found in the full cohort of DLBCL-NOS and HGBCL presenting in the ocular adnexa.

### 2.5. LymphGen Classification

The LymphGen algorithm [7] classified 18 (64%) of the 28 patient samples in our cohort (Figure 2). In DLBCL-NOS patients, the most frequent subtypes were MCD (9 of 26, 35%) and EZB (5 of 26, 19%) (Figure 2). Primary OA-DLBCL-NOS exhibited the same pattern with frequent MCD (5 of 14, 36%) and EZB (3 of 14, 21%) cases as well as one ST2 and one BN2 case (7%) (Figure 2). In relapsed DLBCL-NOS involving the ocular adnexa, MCD cases constituted 50% (3 of 6) (Figure 2). The two primary OA-HGBCL cases were classified as EZB or EZB/ST2 (genetic composite case) (Figure 2).

### 2.6. Survival Analyses

Patients with relapsed DLBCL-NOS were excluded (n = 6), and survival was computed for DLBCL-NOS and HGBCL grouped together (n = 22), as the two patients with HGBCL both had limited stage disease and a favorable outcome, being alive 5 years after diagnosis. Patient survival rates in this cohort included a 5-year overall survival (OS) of 48% (95% CI: 31–75%), a 5-year progression-free survival (PFS) of 44% (95% CI: 27–71%), and a 5-year disease-specific mortality (cumulative incidence) of 28% (95% CI: 11–47%). Patients receiving front-line rituximab-based chemotherapy (11 of 22, 50%) showed a 5-year OS of 73% (95% CI: 51–100%), and a 5-year PFS of 64% (95% CI: 41–99%). None of the patients receiving front-line rituximab-based chemotherapy died from lymphoma-related deaths, and only one patient experienced relapse of lymphoma within 5-years. Patients receiving front-line rituximab-based chemotherapy (n = 11) had a significantly better OS, PFS, and disease-specific mortality in comparison to patients receiving other (n = 8) or no treatment (n = 3), when these two groups were compiled (Table 2, Supplementary Figure S2).

In the full cohort (n = 22) excluding relapsed DLBCL-NOS, no significant associations were found between 5-year survival rates and *MYD88* pathogenic variants, COO, or double-expressor lymphoma (Table 2). The 5-year survival rates of the LymphGen MCD subgroup did not differ significantly from non-MCD cases (EZB, ST2, EZB/ST2, and BN2 compiled) (Table 2). Ann Arbor stage and treatment differed significantly between double-expressor lymphoma patients and non-double-expressor patients (Supplemental Table S1). A significantly lower proportion of the double-expressor lymphoma patients received rituximab-based chemotherapy as compared to the non-double-expressor patients (*p* = 0.009) (Supplemental Table S1). No other clinical characteristics differed significantly between the molecular groups in the survival analyses (Supplementary Table S1). In multivariable Cox regression, both rituximab-based chemotherapy and *MYD88* mutations were significantly (and inversely) associated with OS, whereas only rituximab treatment was associated with PFS (Table 3). However due to low patient numbers, these results need to be interpreted carefully.

## 3. Discussion

In this study, targeted deep sequencing of 128 highly lymphoma-relevant genes was applied to identify pathogenic variants and CNVs in 26 patients with DLBCL-NOS involving the ocular adnexa and 2 patients with HGBCL with *MYC* and *BCL2* rearrangements. The mutational landscape has not been thoroughly explored, and previous targeted NGS studies on ocular adnexal DLBCL-NOS have only included seven and three patients, respectively [21,22]. To the best of our knowledge, this is the most comprehensive genetic study of DLBCL-NOS presenting in the ocular adnexa, and the first study to elucidate genetic variants in ocular adnexal HGBCL.

Primary OA-DLBCL-NOS (n = 14) constituted 54% of DLBCL-NOS cases involving the ocular adnexa and was characterized by a heterogenous genetic landscape with various pathogenic variants and LymphGen genetic subtypes identified. Primary OA-DLBCL-NOS had a relatively high proportion of non-GCB cases (n = 8, 57%) and the LymphGen MCD subtype (n = 5, 36%). The prevalence of patients with the MCD subtype was thus higher in primary OA-DLBCL-NOS compared to the original LymphGen study, where MCD cases constituted 14% [7]. The MCD subtype is associated with the non-GCB COO subtype and is enriched for genetic lesions activating the B-cell receptor (BCR) and Toll-like receptor (TLR) signaling pathways, resulting in increased NF-κB activity including *MYD88* and *CD79B* pathogenic variants [7,8]. Additionally, the genetic features of MCD overlap with those reported in several primary extranodal LBCLs of non-GCB origin and immune-privileged sites [24,25,26,27,28,29,30,31,32,33]. In accordance with previous PCR analyses of *MYD88* in the present cohort [20], we found that primary OA-DLBCL-NOS had a relatively high prevalence of *MYD88* pathogenic variants (n = 4, 29%), and that *CD79B* pathogenic variants were more frequent (n = 3, 21%) than previously detected by PCR [20], possibly due to increased sensitivity by targeted NGS analysis. In a smaller study including six primary OA-DLBCL-NOS patients, *MYD88* pathogenic variants were found in a higher proportion of patients (4 of 6, 67%), which may be attributed to the higher percentage of non-GCB patients in this study compared to our study (83% vs. 57%) [21]. Still, the frequency of pathogenic *MYD88* variants in our cohort of primary OA-DLBCL-NOS is slightly higher compared to DLBCL-NOS located in the lymph nodes (17–20%) [15,25]. This corresponds well to the slightly lower frequency of non-GCB cases in nodal DLBCL-NOS (approx. 40%) [1]. However, the frequency of *MYD88* variants in our cohort of primary OA-DLBCL-NOS does not match that of LBCL of immune-privileged sites (60–78%) [24,25,26,27], or extranodal LBCLs with high non-GCB frequencies, including primary breast DLBCL-NOS (39–56%) [28,29], primary cutaneous DLBCL, leg type (75–79%) [30,31], intravascular LBCL (44–57%) [32,33,34], or primary sinonasal DLBCL-NOS (approx. 50%) [35]. Nevertheless, in primary OA-DLBCL-NOS, we now identify frequent pathogenic variants in several of the genes that either link primary OA-DLBCL-NOS to the MCD subtype and NF-κB activation [7,8], or which are mutated in several of the abovementioned extranodal lymphomas [26,27,28,29,30,31,32,34,35], including *CD79B* (n = 3, 21%), the proto-oncogene *PIM1* (n = 3, 21%), putative tumor suppressor *TBL1XR1* (n = 3, 21%), and putative tumor suppressor *SETD1B* (n = 3, 21%). Several large genetic studies including both nodal and extranodal DLBCL-NOS have reported comparable mutational frequencies in *PIM1* (11–28%), and slightly lower frequencies in *CD79B* (5–15%), *TBL1XR1* (3–13%), and *SETD1B* (8–10%) [6,8,9,10,36].

Although limited by cohort size, we saw a possible link between the genetic profile of patients with primary OA-DLBCL-NOS (n = 14) and patients with relapsed DLBCL-NOS involving the ocular adnexa (n = 6). All six patients with relapsed DLBCL-NOS involving the ocular adnexa were of non-GCB origin, and 50% of patients were assigned to the MCD subtype (n = 3). Relapsed DLBCL-NOS patients carried mutations in several of the same genes as primary OA-DLBCL-NOS, including pathogenic variants in *PIM1* (n = 3), *CD79B* (n = 3), and *MYD88* (n = 2), as well as other genes linked to the MCD subtype: *BTG1* (n = 2), *BTG2* (n = 2), *KLHL14* (n = 2), *ETV6* (n = 2), *IRF4* (n = 2), and *CDKN2A* loss (n = 2) [7,8]. Primary extranodal location was predominant in relapsed DLBCL-NOS patients (4 of 6 patients). Two of the relapsed DLBCL-NOS patients had previous testicular DLBCL with or without nodal involvement and were of MCD subtype in accordance with previous testicular DLBCL studies showing pathogenic variants in MCD defining genes [15,26].

Despite the increased frequency, the MCD subtype does not cover the full genetic spectrum of primary OA-DLBCL-NOS. In this study, the EZB subtype constituted 21% of the primary OA-DLBCL-NOS patients (n = 3 of 14). The EZB subtype is characterized by epigenetic dysregulation and rearrangement of *BCL2* [7,8]. Accordingly, primary OA-DLBCL-NOS of the EZB subtype frequently carried a rearrangement of *BCL2* (n = 2) and *BCL6* (n = 2) as well as pathogenic variants or loss of *TNFRSF14* (n = 2), and pathogenic variants in *POU2F2* (n = 2). As expected, the EZB subtype was also prevalent in primary OA-HGBCL, in which the two patients within this study were classified as either EZB subtype or EZB/ST2 subtype. In addition to the rearrangement of *MYC* and *BCL2,* these patients frequently had pathogenic variants in *CREBBP* (n = 2), *SGK1* (n = 2), and *MYC* (n = 2).

Patient survival in our cohort was associated with treatment, as patients receiving front-line rituximab-based chemotherapy (n = 11) had a superior survival compared to the patient group receiving other treatment (n = 8) or no treatment (n = 3) (Table 2). This was expected as patients receiving “other” treatment mainly received radiotherapy alone (n = 7), either due to comorbidities/frailty (n = 3) or for low-stage DLBCL-NOS (n = 4), and all the patients not receiving any treatment (n = 3) died within 1.5 months after diagnosis. None of the patients receiving front-line rituximab-based chemotherapy (excluding relapsed DLBCL-NOS) died of lymphoma-related deaths in our cohort, and only one patient experienced a relapse of disease within 5 years. This corresponds well to the recently published high survival rates in a cohort of 18 primary OA-DLBCL-NOS patients treated with R-CHOP [37], and limited-stage DLBCL-NOS in general treated with R-CHOP [38]. In univariable survival analyses, neither non-GCB origin, double-expressor lymphoma, LymphGen MCD subtype, or *MYD88* pathogenic variants were significantly associated with 5-year survival rates in our cohort (Table 2). This could be due to the small sample size and heterogenous treatment, as these molecular characteristics have previously been associated with survival in larger DLBCL-NOS studies [7,8,25,39,40,41]. Here it should be noted that a significantly lower proportion of the double-expressor lymphoma patients received rituximab-based chemotherapy compared to the non-double-expressor lymphoma patients (Supplementary Table S1), which introduces a bias in this univariable analysis. By multivariable analysis, we found that OS was associated with both rituximab-based chemotherapy (superior survival) and *MYD88* mutation (inferior survival), whereas only rituximab-based therapy was associated with superior PFS (Table 3).

Incorporation of genetic tumor profiling might be the next step in future precision medicine with the selection of targeted therapy based on specific genetic subtypes and involved biological pathways [42]. In line with this, the first potential evidence of treatment allocation based on the molecular profile recently appeared in DLBCL-NOS [11,12,13]. From this perspective, DLBCL-NOS patients with the MCD subtype might be potential candidates for therapies targeting the BCR pathway and NF-κB signaling such as ibrutinib (Bruton tyrosine kinase inhibitor) or lenalidomide (immune modulator), respectively [7,42,43,44].

Studying a very rare disease poses some limitations. However, for the application of future precision medicine and the translation of molecular DLBCL-NOS trials to ocular adnexal DLBCL-NOS, we argue that it is important to evaluate the genetic landscape in this specific subset of patients. Within the inclusion period (1980–2017), various diagnostic methods have been used for patient work-up and staging, and approximately half of the patients in this study have not been treated with R-CHOP. The restricted cohort size and heterogenicity in treatment modalities over time are inherent limitations of retrospective studies of rare diseases. This may have introduced a bias in the survival analyses and brings an uncertainty of the estimates of the mutational frequencies. Furthermore, the cohort size was limited by a percentage of exclusions (n = 6) compared to our previous study on molecular and genetic characteristics in this cohort [20], which also impacted the survival analyses, as the excluded patients had an inferior survival. This highlights the challenge in molecular analyses of older formalin-fixed, paraffin-embedded (FFPE) tissue, and more comprehensive sequencing analysis (e.g., whole genome sequencing or whole exome sequencing) might have been impeded further by this. In line with this, COO was based on immunohistochemical classification by Hans’ algorithm, which has previously shown good concordance with the results of gene expression profiling (86%) [41]. This approach was chosen as gene expression profiling analysis using older FFPE tissue can be difficult, as we have recently demonstrated in a similar cohort of sinonasal DLBCL-NOS diagnosed between 1980 and 2018 [35]. In this study, COO classification by Nanostring Lymph2Cx gene expression profiling was only feasible in 66% of patients, and thus this approach could have further limited the cohort size in the present study [35].

Although limited by the rarity of the disease and the retrospective design, our study indicates that patients with LBCL involving the ocular adnexa can be subdivided into different genetic subtypes. A relatively large subset of primary OA-DLBCL-NOS patients (36%) exhibited genetic features according to the LymphGen MCD subtype with pathogenic variants in several genes that are involved in NF-κB signaling, and which are recurrently mutated in other primary extranodal DLBCLs of non-GCB origin, including those of immune privileged sites. These findings may prove important for the extension of future therapeutic advancements in DLBCL-NOS to the rare subgroup of ocular adnexal DLBCL-NOS patients.

## 4. Materials and Methods

### 4.1. Study Design and Patient Selection

The study is a retrospective analysis of tumor samples and clinical data of patients diagnosed in Denmark from 1980 to 2017 with LBCL involving the ocular adnexa, i.e., the orbits, conjunctiva, eyelids, lacrimal glands, or lacrimal drainage system. Patients were identified from the previously published Danish cohort of ocular adnexal lymphomas [18,20].

The study included patients with: (1) primary OA-LBCL as defined by localized LBCL involvement of the ocular adnexal region with or without involvement of adjacent structures or regional lymph nodes (Ann Arbor stage IE-IIE) and no prior lymphoma, (2) disseminated LBCL (Ann Arbor stage III-IV) with concurrent involvement of the ocular adnexa at the time of diagnosis, and (3) relapsed LBCL with prior systemic lymphoma and secondary relapse of LBCL to the ocular adnexa. Prior lymphoma included all lymphoma subtypes. However, OA-LBCL patients with a prior diagnosis of untreated SLL bone marrow involvement were not categorized as relapsed LBCL if there was no bone marrow transformation at the time of OA-LBCL diagnosis. Patients with no prior lymphoma diagnosis, but who were not staged at OA-LBCL diagnosis, were categorized as unstaged LBCL.

The manuscript was prepared according to STROBE statement for cohort studies [45].

### 4.2. Clinical Data

Patient clinical data were extracted from the database of our Danish cohort of ocular adnexal lymphomas from 1980 to 2017 [18]. Clinical data in this database have originally been retrieved from clinical records and the Danish Registry of Causes of Death [18]. For the present study, clinical and follow-up information was further updated in 2023 by searching clinical records, or if clinical records were not available, by searching the Danish National Lymphoma Registry [46] and the Danish Registry of Pathology, which includes all biopsy-verified lymphoma relapses. Clinical data included information on date of diagnosis, age, sex, ocular adnexal LBCL location, systemic lymphoma involvement, Ann Arbor staging [47], paraclinical findings including imaging and bone marrow biopsy, treatment, and treatment response. Systemic involvement and staging were based on the diagnostic techniques available at the time of diagnosis. Follow-up included information on progression or relapse of disease, disease stage at last follow-up, survival duration, and cause of death if relevant. Patients who died because of lymphoma progression or complications with treatment were registered as lymphoma-related deaths. In cases where information on cause of death was not available, the patients were registered as dead of unknown cause.

### 4.3. Tumor Samples and Histopathological Classification

Tumor samples from OA-LBCL diagnosis (1980–2017) were retrieved from the archives of Danish pathology departments [20]. All OA-LBCL biopsies were reclassified according to the WHO Classification of Tumors of Haematopoietic and Lymphoid Tissues 5th edition [5] as part of a previously published study [20]. Briefly, this included a morphological evaluation of hematoxylin-eosin stained slides, immunohistochemistry, Epstein–Barr virus (EBV) assessment, double-expressor lymphoma assessment, and evaluation of *MYC*, *BCL2*, and *BCL6* rearrangement [20]. Classification of COO was performed using the Hans’ algorithm, dividing patients into GCB or non-GCB origin with a cutoff value of ≥30% for positively stained tumor cells in CD10, BCL6, and MUM1 [20,23]. Immunohistochemical overexpression of MYC and BCL2 for assessment of double-expressor lymphoma was defined by cutoff values of ≥40% and ≥50%, respectively [20,40]. Epstein–Barr virus status was evaluated by EBV-encoded RNA in situ hybridization (EBER-ISH), and *MYC*, *BCL2,* and *BCL6* gene rearrangements were analyzed by fluorescence in situ hybridization (FISH) [20]; see Supplementary Methods.

### 4.4. Targeted Next-Generation Sequencing

Mutational analysis was performed on FFPE tumor samples from the time of OA-LBCL diagnosis (1980–2017) with the validated BLYMFv2-targeted NGS Ion-Torrent-based AmpliSeq panel, as previously extensively described [48]. The BLYMFv2 panel is an updated version of the previously published diagnostic LYMFv1 panel, where genes have been selected based on a comprehensive literature search (~300 articles) of frequencies and clinical relevance of genetic alterations in B-cell lymphomas [48]. The BLYMFv2 panel covers 128 B-cell lymphoma-relevant genes and contains 3359 amplicons divided in two primer pools; see Supplementary Table S2 for the list of genes. The BLYMFv2 panel’s variant calling quality was validated through comparison with whole exome sequencing and LYMFv1 data from previously sequenced samples.

Genomic DNA was isolated from ocular adnexal FFPE tumor biopsies, followed by preparation of the BLYMFv2 libraries and sequencing on the Ion S5^TM^ Sequencing platform (Thermo Fisher Scientific, Waltham, MA, USA), as previously described [48]. In brief, library-PCR was performed on all samples according to manufacturer’s procedures, followed by FuPa digestion of the primers, barcoding of the library-DNA, purification, and consecutive normalization; see Supplementary methods for detailed description. In case samples failed targeted NGS because of high rates of deamination artefacts (G > A/C > T), an Uracil-DNA glycosylase repair kit (Thermo Fisher Scientific, Waltham, MA, USA) was used, and libraries were re-prepared and re-run as normal, according to the manufacturer’s protocol. Furthermore, samples were excluded if the transition to transversion ratio was ≥5, as this indicates formalin-fixation-induced artefacts, and if the average read count of the sample was below 100 reads.

The sequencing reads were aligned to the human reference genome (GRCh37/hg19) with the TMAP 5.0.7 software (default parameters, https://github.com/iontorrent/TS (accessed on 1 June 2018)). The Torrent Variant Caller (Thermo Fisher Scientific, Waltham, MA, USA) called all variants. The threshold for calling variants was a coverage of ≥100 reads and a variant allele frequency of ≥10%. As previously described [48], variants with a population frequency of more than 1% in the 1000 Genomes Project, or variants that appear in three DNA mixtures of ‘healthy’ individuals (n = 288; sequenced during validation [48]) were excluded from further analysis, in addition to variants with a high strand bias (>90%) and sequencing artifacts induced by homopolymeric regions. All remaining variants were classified according to pathogenicity as previously described [48]. Briefly, this included annotation of variants in the Geneticist Assistant NGS interpretive Workbench (SoftGenetics version 1.8.1) into class 1 (benign), class 2 (likely benign), class 3 (unknown significance), class 4 (likely pathogenic), or class 5 (pathogenic) [49]. Class 4 and 5 were classified as pathogenic variants. Additionally, class 3 variants were classified as pathogenic if they exhibited a high Combined Annotation-Dependent Depletion (CADD)–PHRED score (>25) [50], or in case of a CADD-PHRED score between 10 and 25 with ≥2 additional pathogenic notations in the prediction tools SIFT, Polyphen2_HDIV, LRT, and MutationTaster [48]. Only variants classified as pathogenic are presented in the oncoprint plots (Figure 2 and Supplementary Figure S1).

Copy Number Variation (CNV) analysis was performed on the targeted NGS data, by computing the normalized median base coverage per amplicon of all included genes. Samples with high variability or low quality were excluded, whereafter a total of 28 samples from three different sequencing runs were analyzed. The amplicon coverage per sample was normalized with all samples included in the run to determine the CNVs (gains or losses) per gene. Gains and losses were called when more than two consecutive amplicons were above or below the 99% confidence intervals of the respective amplicons from the normalized data of all genes, except for *CDKN2A* for which the 95% confidence interval was used [31,51]. The confidence interval of *CDKN2A* was modified because the normalized amplicon coverage of *CDKN2A* can be influenced by the frequent loss of this gene in DLBCL-NOS, as previously reported in the literature [52].

### 4.5. LymphGen Classification

LymphGen classification [7] was applied to categorize the patients into genetic subtypes by adding data on all variants (including variants of unknown pathogenicity and benign variants) and rearrangement status of *BCL2*, and *BCL6* for cluster allocation. Tumors with subtype probabilities of >50% were included as subtype members, including both extended (>50–90%) and core subtype members (>90%) [7]. CNV results were not included in the LymphGen classification as our panel only provides limited CNV data. However, the LymphGen algorithm has been designed to function using various combinations of genetic data including both whole-exome and targeted sequencing, and permits the use of mutation-only data (without the identification of the A53 subtype as this cluster allocation relies on CNV data) [7]. Furthermore, the LymphGen algorithm has been validated on genetic data from other cohorts and FFPE tissue [12,53].

### 4.6. Statistics

Pathogenic variants and CNV results were visualized by OncoPrint plots. Fisher’s exact test and the Mann–Whitney U test were applied for comparing categorical variables and non-parametric continuous variables between subgroups, respectively. Patient 5-year survival was calculated for all LBCL cases, with the exclusion of relapsed LBCL involving the ocular adnexa. OS was defined as the time from OA-LBCL diagnosis to death of any cause. PFS was defined as the time from OA-LBCL diagnosis to either progression, relapse, or death from all causes. Disease-specific mortality was defined as the time from OA-LBCL diagnosis to death related to lymphoma, with death from other and unknown causes as competing risk. Patients were administratively censored after 5 years of follow-up or censored at last follow-up if an event had not occurred. Survival was estimated using the Kaplan–Meier and cumulative incidence curves with competing risk. Kaplan–Meier curves for OS and PFS were compared using the log-rank test, and corresponding hazard ratios (HR) and 95% confidence intervals (95% CI) were calculated with Cox proportional hazards regression. The proportional hazard assumption was tested for all covariates, and no violations were found. Cumulative incidence curves for disease-specific mortality were compared by Gray’s test, and HR and 95% CI were calculated both by competing risk regression (Fine and Gray) and Cox proportional hazard regression by censoring patients dying from other/unknown causes (cause-specific hazard method). *p*-values <0.05 were considered significant. Statistical analyses were performed with R software (packages: “readxl”, “tidyverse”, “survival”, “survminer”, “ggsurvfit”, “gtsummary”, “tidycmprsk”, “data.table”, “ComplexHeatmap”, “tableone”; R version 4.2.2, R Core Team 2022) and R Studio (version 2022.12.0.353, Posit team 2022).

## Figures and Tables

**Table 2:** Univariable 5-year survival correlations of 22 patients with large B-cell lymphoma involving the ocular adnexa.

		Overall Survival				Progression-Free Survival				Disease-Specific Mortality						
		Log-Rank	Cox Regression			Log-Rank	Cox Regression			Gray’s Test	Cox Regression			Competing Risk Regression		
	n	*p*	HR	95% CI	*p*	*p*	HR	95% CI	*p*	*p*	HR	95% CI	*p*	HR	95% CI	*p*
*MYD88* mutation	6	0.12	2.70	0.76–9.62	0.13	0.24	2.04	0.59–6.99	0.26	0.66	1.62	0.29–8.97	0.58	1.45	0.31–6.87	0.64
LymphGen MCD	6	0.12	3.19	0.68–15.0	0.14	0.12	3.19	0.68–15.0	0.14	0.88	1.25	0.08–20.0	0.88	1.25	0.10–16.4	0.87
Cell-of-origin: non-GCB	9	0.85	1.11	0.34–3.65	0.86	0.86	0.90	0.28–2.83	0.85	0.59	0.64	0.12, 3.52	0.61	0.64	0.13–3.01	0.57
Double-expressor	11	0.083	2.86	0.82–9.94	0.098	0.20	2.12	0.66–6.77	0.21	0.059	6.34	0.73–54.7	0.093	5.98	0.71–50.4	0.10
Rituximab-based chemotherapy	11	**0.0089**	0.19	0.05–0.74	**0.017**	**0.023**	0.26	0.08–0.89	**0.031**	**0.005**	NA	NA	NA	NA	NA	NA

*MYD88* mutation: Survival of patients with pathogenic variants of *MYD88* was compared to *MYD88* wild-type patients (reference, HR = 1); LymphGen MCD: survival of patients with LymphGen MCD subtype is compared with non-MCD patients (all classified cases grouped together) (reference, HR = 1); Cell-of-origin: survival of non-germinal center B-cell (non-GCB) patients are compared to GCB patients (reference, HR = 1); Double-expressor: survival of double-expressor lymphoma patients with immunohistochemical overexpression of both MYC and BCL2 are compared to non-double-expressor patients. Rituximab: survival of patients receiving front-line rituximab-based chemotherapy is compared to patients receiving other (n = 8) or no treatment (n = 3) (reference = non-rituximab group, HR = 1). Abbreviations: HR: hazard ratio; 95% CI: 95% confidence interval; NA: not applicable.

**Table 3:** Multivariable Cox regression in 22 patients with large B-cell lymphoma involving the ocular adnexa.

		5-Year Overall Survival			5-Year Progression-Free Survival		
	**n**	**HR**	**95% CI**	***p***	**HR**	**95% CI**	***p***
Rituximab	11	0.14	0.03–0.59	**0.008**	0.21	0.06–0.77	**0.019**
*MYD88* mutation	6	4.66	1.08–20.1	**0.039**	2.90	0.76–11.0	0.12

*MYD88* mutation: survival of patients with pathogenic variants of *MYD88* was compared to *MYD88* wild-type patients (reference, HR = 1); rituximab: survival of patients receiving front-line rituximab-based chemotherapy is compared to patients receiving other (n = 8) (chemotherapy and/or radiotherapy) or no treatment (n = 3) (reference = non-rituximab group, HR = 1). Abbreviations: HR: hazard ratio; 95% CI: 95% confidence interval.

## Data Availability

The data presented in this study are available on request from the corresponding author. The data are not publicly available due to Danish Data Protection legislation.

## Supplementary Materials

The supporting information can be downloaded at: https://www.mdpi.com/article/10.3390/ijms25063094/s1. References [54,55] are cited in the supplementary materials.
