# Supplementary material for: The Genetic Profile of Large B-Cell Lymphomas Presenting in the Ocular Adnexa
Source: Int J Mol Sci. 2024 Mar 7;25(6):3094. doi: 10.3390/ijms25063094 (PMC10970350; doi:10.3390/ijms25063094)
Supplement: Supplementary file 1 [file ijms-25-03094-s001.zip › Supplementary Figure S1 including figure caption.pdf]

Supplementary Figure S1

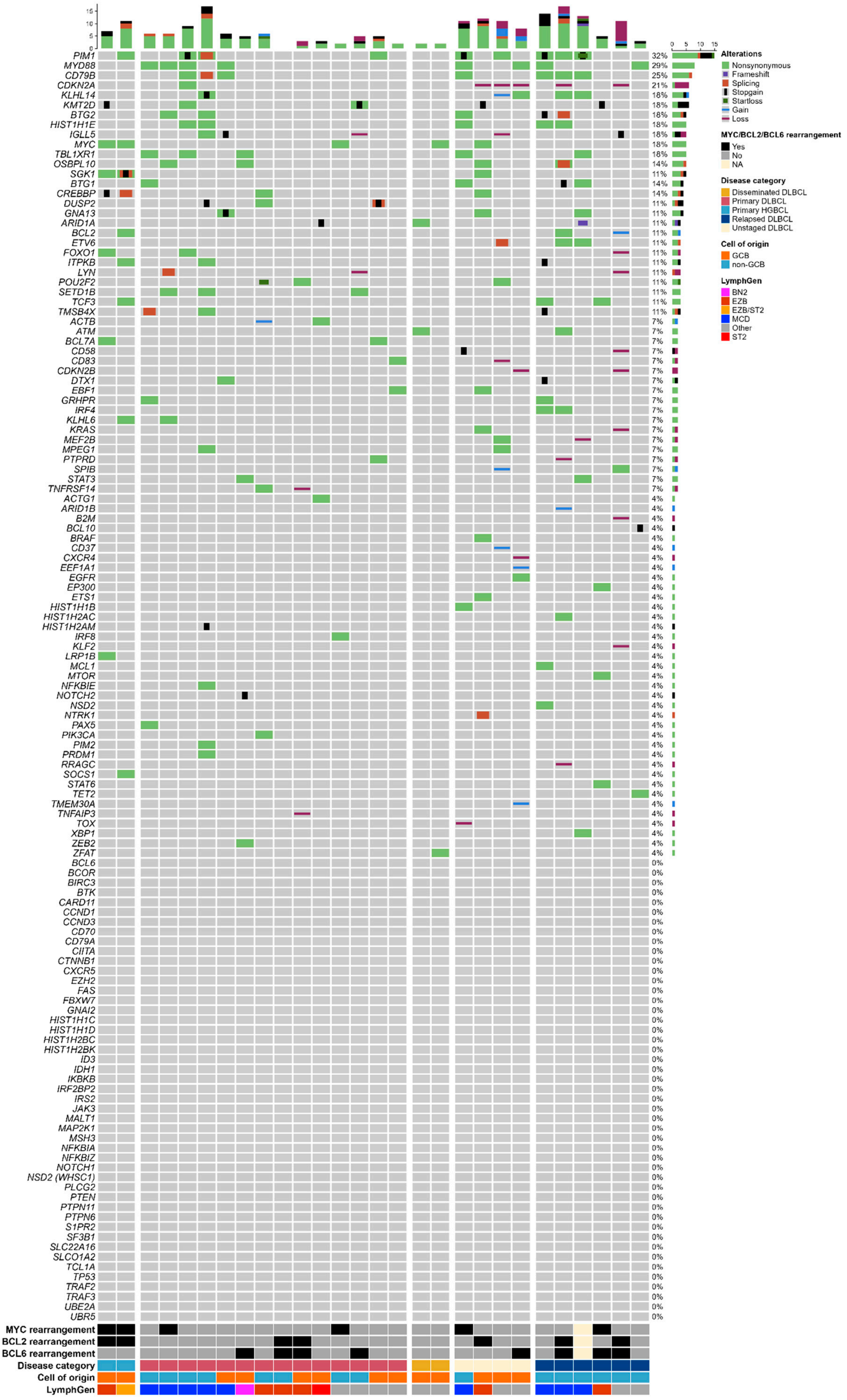

Supplementary Figure S1: Oncoprint including all sequenced genes in 28 LBCL patients with ocular adnexal involvement. Every row represents a specific gene or clinical/molecular characteristic, and every column represents a patient. The percentage of patients with alterations in the denoted gene is shown on the right side. The number of gene alterations per patient is shown at the top. Rearrangements of *MYC*, *BCL2*, and *BCL6* as well as disease category, cell-of-origin by Hans' classification, and LymphGen genetic subgrouping are shown at the bottom. Patients are sorted in the same order as Figure 2.

DLBCL: diffuse large B-cell lymphoma; GCB: germinal center B-cell; HGBCL: high-grade B-cell lymphomas with *MYC* and *BCL2* rearrangements; NA: Not available
