# Supplementary material for: The Genetic Profile of Large B-Cell Lymphomas Presenting in the Ocular Adnexa
Source: Int J Mol Sci. 2024 Mar 7;25(6):3094. doi: 10.3390/ijms25063094 (PMC10970350; doi:10.3390/ijms25063094)
Supplement: Supplementary file 1 [file ijms-25-03094-s001.zip › Supplementary Figure S2 including figure caption.pdf]

Supplementary Figure S2

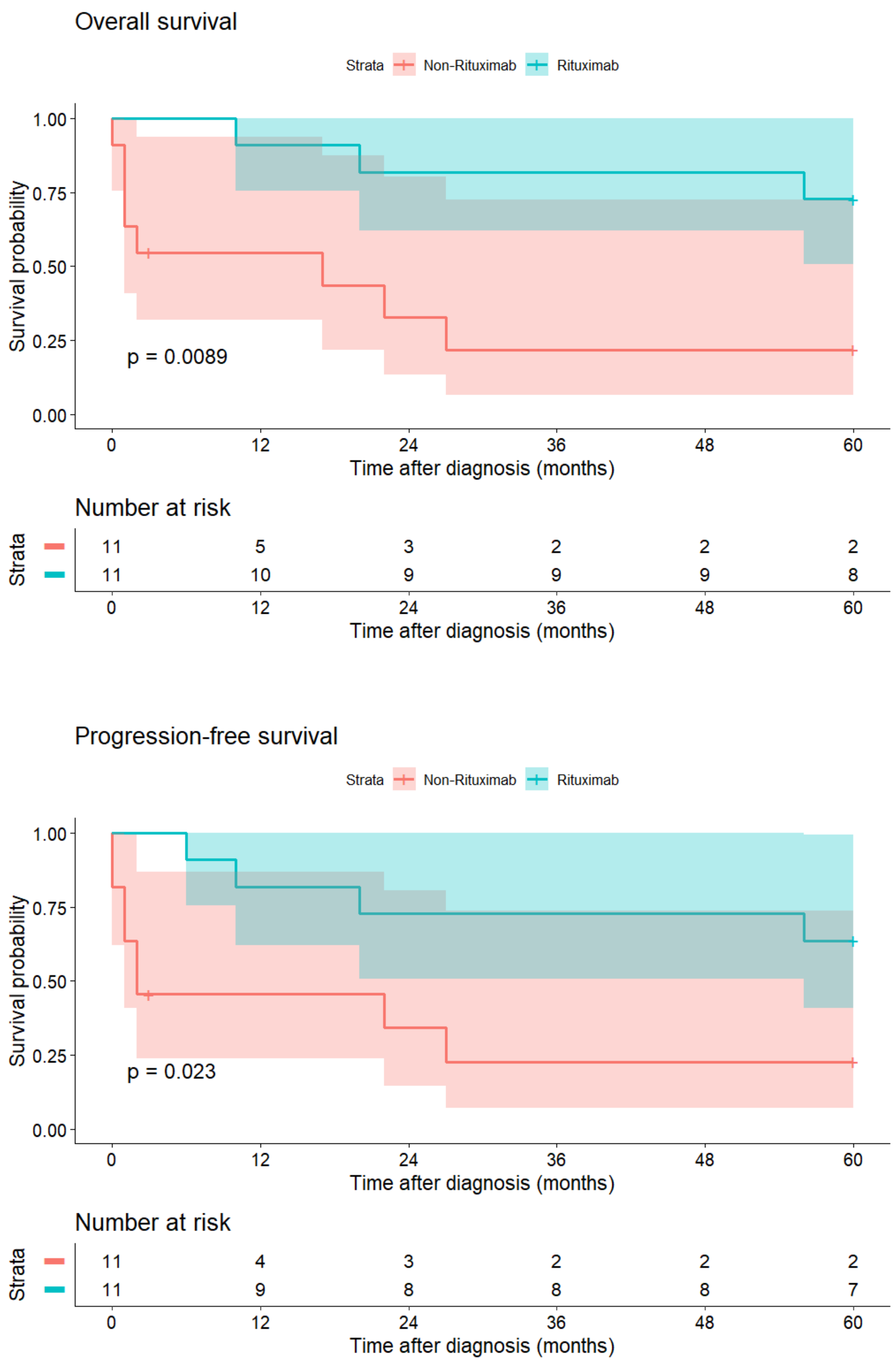

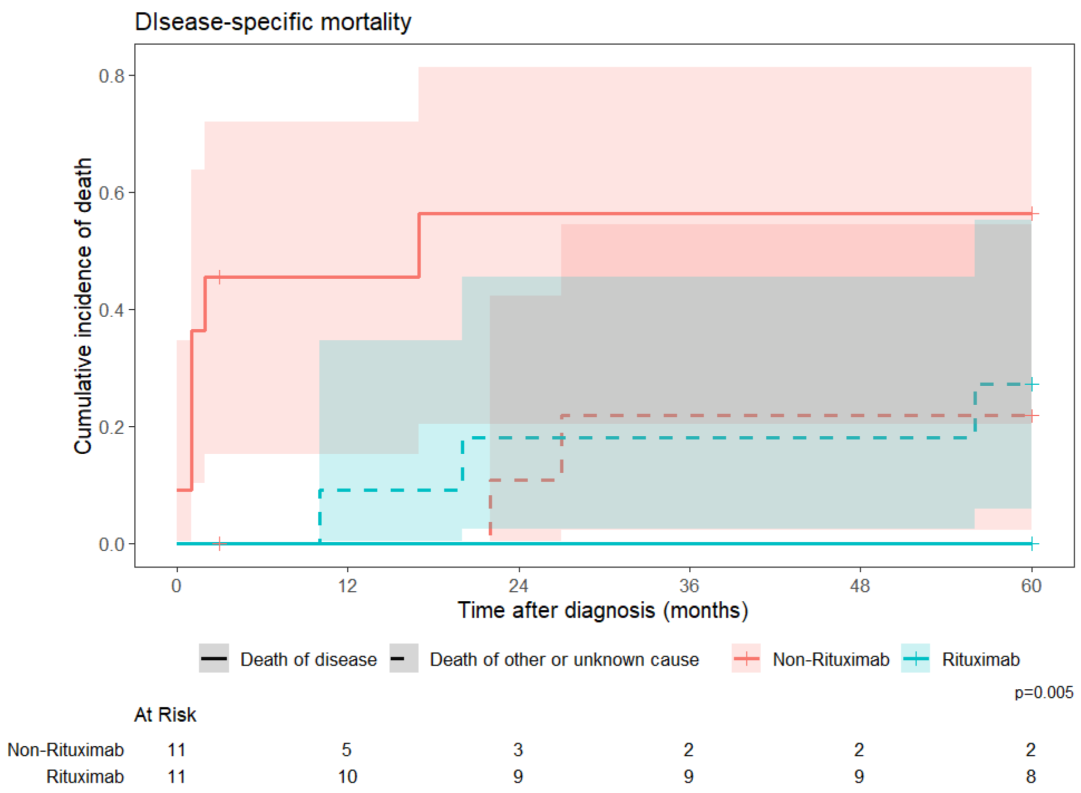

Supplementary Figure S2: Survival of patients receiving Rituximab-based chemotherapy (n=11) as compared to patients receiving other (n=8) (radiotherapy and/or chemotherapy) or no treatment (n=3) (non-Rituximab group). Overall survival, progression-free survival, and disease-specific mortality was significantly better in patients receiving Rituximab-based chemotherapy as compared to other or no treatment. None of the patients receiving Rituximab-based chemotherapy died from lymphoma-related deaths within follow-up. 95% confidence intervals are included. Patients with relapsed DLBCL-NOS were excluded from the survival analyses.
