# Supplementary material for: The Genetic Profile of Large B-Cell Lymphomas Presenting in the Ocular Adnexa
Source: Int J Mol Sci. 2024 Mar 7;25(6):3094. doi: 10.3390/ijms25063094 (PMC10970350; doi:10.3390/ijms25063094)
Supplement: Supplementary file 1 [file ijms-25-03094-s001.zip › Supplementary methods.pdf]

Supplementary Methods

Fluorescence in situ hybridization (FISH):

As part of a previously published study [1], *MYC*, *BCL2,* and *BCL6* gene rearrangements were analyzed by fluorescence in situ hybridization (FISH) with break apart probes in accordance with the guidelines provided by the manufacturer (Zytovision, Bremerhaven, Germany), utilizing the HYBrite platform (Abbott Molecular). One case was additionally examined with the *MYC/IGH* dual-fusion probe (Zytovision). Following the hybridization process, the nuclei were counterstained with DAPI II (ZytoVision). A total of one hundred nuclei were assessed for each sample, employing a positive threshold of 10%. Only nuclei exhibiting complete visibility of the nuclear membrane were included in the assessment.

DNA extraction

Genomic DNA was isolated from FFPE biopsies either with the QIAamp DNA micro kit (n=30) (Qiagen, Venlo, the Netherlands) as described previously [1,2], or by automatic isolation from 10 µm sections with the Tissue Preparation System (TPS) robot (n=3) (Siemens Healthcare Diagnostics, Erlangen, Germany) as previously described [3]. DNA concentrations were quantified with the Qubit™ dsDNA HS Assay Kit (Invitrogen, Waltham, MA, USA).

Targeted next-generation sequencing:

Mutational analysis was performed as previously described [4]:

> DNA libraries of the BLYMFv2 panel were manually prepared. For PCR, 4 µL with a concentration of optimally 4ng/µL DNA was mixed with 2µL of 5x Ion AmpliSeq HiFi Mix (Ion AmpliSeq™ Library Kit 2.0; ThermoFisher Scientific 4480441, Waltham, MA, USA) and 5 µL of 2x BLYMFv2 Ion AmpliSeq primer pool (ThermoFisher Scientific). The Bio-Rad C1000 Thermal Cycler was used for amplification with the following steps: 99 °C for 2 minutes, 18 cycles of 99 °C for 15 seconds, and 60 °C for 16 minutes.
>
> Following the PCR procedure, the two primer pools were combined and consecutively these primers were digested by adding 1 µL of FuPa reagent (Ion AmpliSeq™ Library Kit 2.0) and 1 µL of water with heating in the Thermal Cycler at 50 °C for 10 minutes, 55 °C for 10 minutes, and 60 °C for 20 minutes. After digestion of the primers, samples were barcoded by adding 2 µL of the barcode primers (Ion AmpliSeq™ Library Kit 2.0), 4 µL of switch solution (Ion AmpliSeq™ Library Kit 2.0) and 2 µL of DNA ligase (Ion AmpliSeq™ Library Kit 2.0), and heated at 22 °C for 60 minutes and 72 °C for 10 minutes.
>
> After barcoding, the sample libraries were manually purified using 50 µL low TE solution and 45 µL AMPure XP beads (Beckman Coulter, Brea, CA, USA; A63882), according to the library purification protocol. Following purification, qPCR was performed using 5 µL of Ion Library TaqMan qPCR Master Mix (2x) and 0.5 µL of Ion Library TaqMan Quantitation Assay (20x; Ion Library TaqMan™ Quantitation Kit; ThermoFisher Scientific; 4468802) with 4.5 µL of 100 times diluted sample purified library. qPCR was performed together with a calibration curve of 10-, 100-, and 1000-times diluted *E. coli* (68 pM), on a Bio-Rad S1000 thermal Cycler with the following settings: 50 °C for 2 minutes, 95 °C for 20 seconds, followed by 32 cycles of 95 °C for 3 seconds and 60 °C for 20 seconds. Based on the outcomes of the qPCR, samples were normalized and pooled, and another qPCR was performed with similar conditions to determine the pool concentration. Libraries were pooled to a final

concentration of 60 pM. These pools were used for sequencing preparation with the Ion Chef^{TM} instrument (ThermoFisher Scientific; 4484177) with the Ion 540^{TM} Chip Kit (ThermoFisher Scientific; A27765). Sequencing was performed with the Ion S5^{TM} Sequencing platform (ThermoFisher Scientific; A27212), according to the manufacturer's protocol (ThermoFisher Scientific).

References

1 Kirkegaard MK, Minderman M, Sjö LD, *et al.* Prevalence and prognostic value of MYD88 and CD79B mutations in ocular adnexal large B-cell lymphoma: a reclassification of ocular adnexal large B-cell lymphoma. *Br J Ophthalmol* Published Online First: 27 October 2021. doi:10.1136/bjophthalmol-2021-319580

2 Kirkegaard MK. Ocular adnexal lymphoma: Subtype-specific clinical and genetic features. *Acta Ophthalmol* 2022;**100**:3–37. doi:10.1111/AOS.15248

3 van Eijk R, Stevens L, Morreau H, *et al.* Assessment of a fully automated high-throughput DNA extraction method from formalin-fixed, paraffin-embedded tissue for KRAS, and BRAF somatic mutation analysis. *Exp Mol Pathol* 2013;**94**:121–5. doi:10.1016/J.YEXMP.2012.06.004

4 de Groen AL, van Eijk R, Bohringer S, *et al.* Frequent mutated B2M, EZH2, IRF8, and TNFRSF14 in primary bone diffuse large B-cell lymphoma reflect a GCB phenotype. *Blood Adv* 2021;**5**:3760–75. doi:10.1182/BLOODADVANCES.2021005215
