# Supplementary material for: The Genetic Profile of Large B-Cell Lymphomas Presenting in the Ocular Adnexa
Source: Int J Mol Sci. 2024 Mar 7;25(6):3094. doi: 10.3390/ijms25063094 (PMC10970350; doi:10.3390/ijms25063094)
Supplement: Supplementary file 1 [file ijms-25-03094-s001.zip › Supplementary tables.pdf]

Supplementary Table S1

Table S1: Comparison of clinical characteristics between molecular groups in the survival cohort (n=22) excluding relapsed DLBCL-NOS cases

	MYD88 Mutation	MYD88 WT	*P*	LymphGen MCD	LymphGen Non-MCD[3]	*P*	Non-GCB	GCB	*P*	Double-expressor	Non double-expressor	*P*
**Number**	6	16		6	8		9	13		11	11	
**Age[1] (years)**	76 (73-91)	74 (70-80)	0.40	76 (73-82)	78 (70-83)	0.95	74 (73-83)	75 (67-79)	0.57	76 (73-89)	72 (65-79)	0.14
**Sex**			1			0.28			0.42			0.67
Male	3 (50.0)	7 (44)		4 (67)	2 (25)		3 (33)	7 (54)		6 (55)	4 (36)	
Female	3 (50.0)	9 (56)		2 (33)	6 (75)		6 (67)	6 (46)		5 (45)	7 (64)	
**Ann Arbor stage**			0.57			1			0.49			**0.045**
IE-IIE	4 (67)	12 (75)		5 (83)	7 (87.5)		8 (89)	8 (62)		7 (64)	9 (82)	
IV	0 (0)	2 (12.5)		0 (0)	0 (0)		0 (0)	2 (15)		0 (0)	2 (18)	
Unstaged	2 (33)	2 (12.5)		1 (17)	1 (12.5)		1 (11)	3 (23)		4 (36)	0 (0)	
**Treatment[2]**			1			1			1			**0.009**
Rituximab + chemotherapy ± RT	3 (50)	8 (50)		3 (50)	5 (63)		5 (56)	6 (46)		2 (18)	9 (82)	
Other/no treatment	3 (50)	8 (50)		3 (50)	3 (37)		4 (44)	7 (54)		9 (82)	2 (18)	
RT ± prednisolone	2 (33)	5 (31)		3 (50)	1 (12.5)		4 (44)	3 (23)		6 (55)	1 (9)	
Chemotherapy ± RT	0 (0.0)	1 (6)		0 (0)	1 (12.5)		0 (0)	1 (8)		1 (9)	0 (0)	
No treatment	1 (17)	2 (13)		0 (0)	1 (12.5)		0 (0)	3 (23)		2 (18)	1 (9)	

1: Displayed as median and interquartile range

2: Statistical significance is tested between the Rituximab-based chemotherapy group and the other/no treatment group, which compiles the following treatment groups: RT ± prednisolone, Chemotherapy ± RT, No treatment

3: The LymphGen non-MCD group includes all other LymphGen subtypes (only classified cases)

Abbreviations: RT: radiotherapy; GCB: germinal center B-cell

Supplementary Table S2

Table S2: Genes included in BLYMFv2 panel							
ACTB	ACTG1	ARID1A	ARID1B	ATM	B2M	BCL10	BCL2
BCL6	BCL7A	BCOR	BIRC3	BRAF	BTG1	BTG2	BTK
CARD11	CCND1	CCND3	CD37	CD58	CD70	CD79A	CD79B
CD83	CDKN2A	CDKN2B	CIITA	CREBBP	CTNNB1	CXCR4	CXCR5
DTX1	DUSP2	EBF1	EEF1A1	EGFR	EP300	ETS1	ETV6
EZH2	FAS	FBXW7	FOXO1	GNA13	GNAI2	GRHPR	HIST1H1B
HIST1H1C	HIST1H1D	HIST1H1E	HIST1H2AC	HIST1H2AM	HIST1H2BC	HIST1H2BK	ID3
IDH1	IGLL5	IKBKB	IRF2BP2	IRF4	IRF8	IRS2	ITPKB
JAK3	KLF2	KLHL14	KLHL6	KMT2D	KRAS	LRP1B	LYN
MALT1	MAP2K1	MCL1	MEF2B	MPEG1	MSH3	MTOR	MYC
MYD88	NFKBIA	NFKBIE	NFKBIZ	NOTCH1	NOTCH2	NSD2 (WHSC1)	NTRK1
OSBPL10	PAX5	PIK3CA	PIM1	PIM2	PLCG2	POU2F2	PRDM1
PTEN	PTPN11	PTPN6	PTPRD	RRAGC	S1PR2	SETD1B	SF3B1
SGK1	SLC22A16	SLCO1A2	SOCS1	SPIB	STAT3	STAT6	TBL1XR1
TCF3	TCL1A	TET2	TMEM30A	TMSB4X	TNFAIP3	TNFRSF14	TOX
TP53	TRAF2	TRAF3	UBE2A	UBR5	XBP1	ZEB2	ZFAT
